# Foundered

**Published:** 1876-04

**Authors:** P. H. Hayes


### FOUNDERED.

P. H. HAYES, M. D.

A very intelligent professor, once in the College at Beirut in Syria, told me that in all his life time he could not remember to have eaten as many melons as he wanted, until in that land, the home of the melon, he finally became possessor of a garden and planted it literally with melons, and when they were fully ripe he sat down one day in the midst of the luscious fruit and ate and ate until he loathed the taste and the odor of melons, and from that day to this, now many years, he never tastes of melons.

A young man, excessively fond of honey, went out with his uncle to cut a bee-tree. When the tree fell, much of the honey was broken up and lay in fragments of comb around the broken hive in the prostrate tree. My informant says he ate at first from his love of the honey, and afterward from the feeling that otherwise it was to be wasted, and finally he became very sick, and has ever since kept clear of honey.

A bright young girl went to a sugar party, one of a little coterie of invited guests. She ate of the sugar in all its forms of wax and granulation until finally coming to an older sister, she says: "Laura, this sugar tastes bitter."

Old Burton, in his "Anatomy of Melancholy," would say of all these, they are "*foundered.*" This very word we use in reference to animals who break through to rich grain and eat until they are surfeited, and often die—they are not so *tough* as humanity—or if they do not, they are stiffened in their limbs, and sometimes for a life-time.

In my last article for the BISTOURY, I said that *exhaustion* is our national disease, and that the breathing of bad air in our homes and offices, was a most prominent and disgraceful cause of it. I also said in that article, that when a great natural law of life was thus broken it would in some way lead to the breaking of other great principles of health, and I may here add that each law thus broken intensifies the evil effects of breaking every other in turn, and that the breaking of one makes it more likely that we shall break many others—nay, almost creates a necessity for doing it. Don't forget that the converse is also most happily true, that if we keep one we are more likely to keep all natural principles of health, and that it becomes a luxury to do so.

I meet many men and women, invalided, "*foundered,*" by excess in hearty foods. They have been too much shut up in the close atmosphere of homes, and this very confinement dulls the palate, and this calls in turn for rich foods and sharp sauces and stimulating drinks by which appetite is stimulated beyond the wants of nature, and the system is loaded up with a quantity and quality impossible to be thoroughly digested. The flushed and heated face testify to the partial fever thus produced, and to the combustion going on within.

I know the partially-foundered man as soon as I meet him. He walks slow; his wind is short; hurry him and his face is fiery red and his whole frame trembles. As soon as this passes he is pale and exhausted. Eating enough for two men he is nevertheless very weak, nay, remember he is weak *because* he eats so much. How many people freely load up their stomachs, never asking how nature is to unload. But nature *must* unload, and it is this very effort to unload which tires and exhausts and makes these persons weak. Even excess in drinking pure water weakens the body, apparently, because nature must set to work to eliminate it somehow from the body. I know what these patients will say when they come to the doctor. They will tell you first of their "*bilious attacks*," really *indigestions*, when the system completely foundered, revolts against all food, and the stomach partially paralyzed sends back the food the way it came, and the head aches enough to "*split*." They will tell you of backache, aching in the nape of the neck, of pains in the joints and in the feet. These are often swollen, red and tender, and as age advances are liable to serious disease. The "peccant humors" engendered from lusts at the table are liable to settle here in the form of *gout* and *senile gangrene*.

I meet many excellent people who are foundered but do not know it. They are on the high road to *Apoplexy*, *Brights' Disease*, *Gout*. The great excess in the use of fats, oils, sugars and starch, *hydro carbons*, in foods, is deposited in a lax, flabby fat upon the exterior of the body, and this is nature's way of getting rid of these. But there is no such convenient way of getting rid of excess in flesh foods. The excess here is so largely thrown upon the kidneys that they finally begin to fail and a partial—*very* partial it may be at first — blood-poisoning—*uremia*—is the consequence.

Curiously enough, whatever thus poisons the blood causes it to circulate the more slowly and feebly, which the very *aspect* of these patients betrays. For at the first, though their faces be red yet they are a *dusky* red, and afterwards when they are pale it is a peculiar *dusky* pallor. Pure blood stimulates the extreme vessels, poisoned blood paralyzes them. The heart works hard to overcome the difficulty, and these patients have always some heart thumpings and pre-cordial distresses.

But I must not forget that the sufferings of animal nature, gross and appaling as they often are from excess, are not to be compared to the agitations and depressions of the mind, the irritability and petulance of tempers, the lights and the darknesses of their moods. While the fever of the feast burns, all goes well, they are hilarious, generous, easy to be entreated. When the fire goes down and the ashes only are left, they are selfish, quickly angry, sometimes half-maniacal with rage. All effort is paralyzed. In all excess there is weakness, exhaustion. The very haste in temper, the sudden mental agitation, the quick alternations of light and darkness in the mind are all evidences of exhaustion and weakness. When we come to treat these patients, we find their show of strength is a cheat. Their muscles are small, their circulation really weak. Their minds are heavy, unfit for concentration and consecutive thinking. Paul says, "I keep my *body* under." The animal soul must not oppress the rational soul.

---

A profitable industry in the vicinity of Cape May, New Jersey, is the mining of ancient cedar-logs in the mire of the swamps. In these swamps, says the Monmouth *Democrat*, are buried enormous trees, at a depth of from three to ten feet. The logs lie one across another, and there is abundant evidence that they are the growth of successive forests. The mode of searching for the logs is as follows: An iron rod is thrust into the soft mud, over which often, the water lies. After several soundings, the workman is able to tell how the tree lies, which is its root-end, and how thick it is. He then contrives to get a chip from the tree, and so determines at once whether it is worth the labor of mining. A pit is now dug, into which the water soon flows, filling it up. The tree is then cut across with a saw at regular intervals, each section floating to the surface. A layer of such trees is found covered by another layer, and these again by another, and even a third, while living trees may still be growing over all.

---

☞ Subscribe for the BISTOURY, and receive for fifty-five cents a year, more valuable hints and recipes, both for the professional reader and for laymen, than is afforded in any other journal, for four times that sum!

